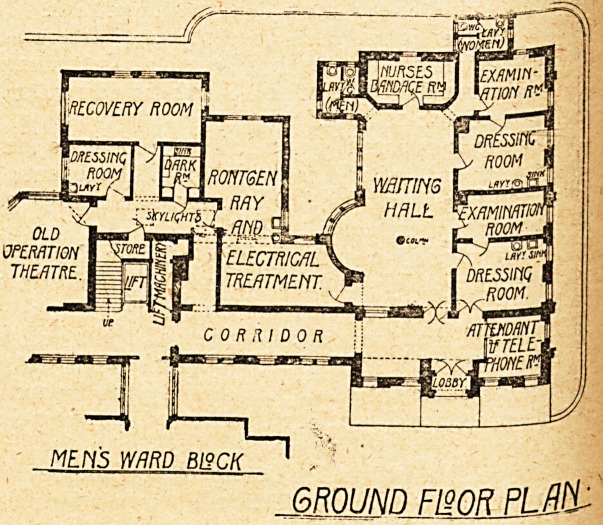# The Royal Gwent Hospital, Newport, Mon.

**Published:** 1917-03-24

**Authors:** 


### HOSPITAL ARCHITECTURE AND CONSTRUCTION.

### The Royal Gwent Hospital, Newport, Mon.

The new wing, the plans of which we give to-day, is three storeys in height, and comprises on the ground floor a complete casualty department, on the first floor a complete ward unit, and on the second floor a complete operation unit. The casualty department includes a large waiting-hall, off which are two examination-rooms, a nurses' bandaging-room, a room for attendant and telephone, and separate sanitary offices for each sex. Adjoining the waiting-hall, but communicating also with the hospital, is a large room for $x$-ray and electrical treatment, formerly the casualty-room; close by is the dark-room. The old operation theatre is now used for minor operations, and has attached to it a dressing-room and large recovery-room.

On the first floor is a men's ward for eighteen beds, with the sanitary offices in a cut-off wing at the north-west end. Adjoining is the ward, kitchen day-room, and bath-room and lavatory.

On the second floor are two operation theatres, with a common sterilising and wash-up room and an anæsthetic-room, which communicates directly with one theatre but not with the other. A large surgeons' room is provided, but for what use exactly is not clear. Adjoining is a surgeons' cloak-room and lavatory and a w.c. There is a large surgical store-room, which, we presume, will be also the theatre-sister's room. It seems a pity that in an otherwise excellently arranged unit the anæsthetic-room could not have been so planned as to communicate with each theatre.

The architect for these alterations was Mr. Henry J. Griggs, A.R.I.B.A., of Newport.

THE ROYAL GWENT HOSPITAL, NEWPORT, MON. (EXTENSIONS).

## Figures and Tables

**Figure f1:**
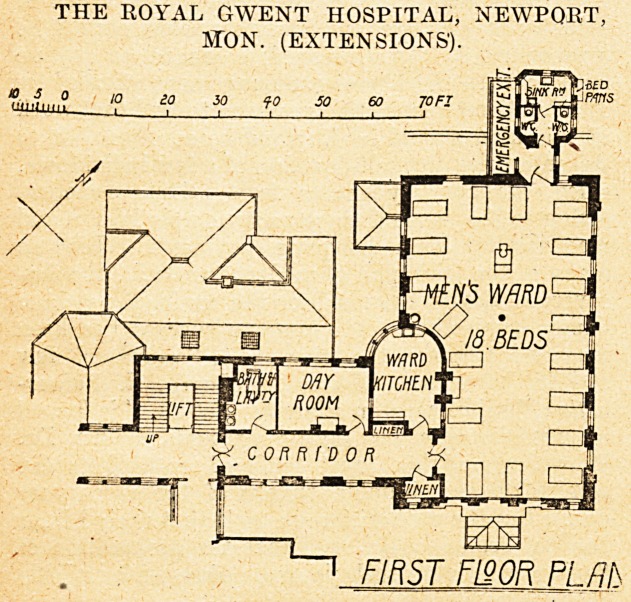


**Figure f2:**
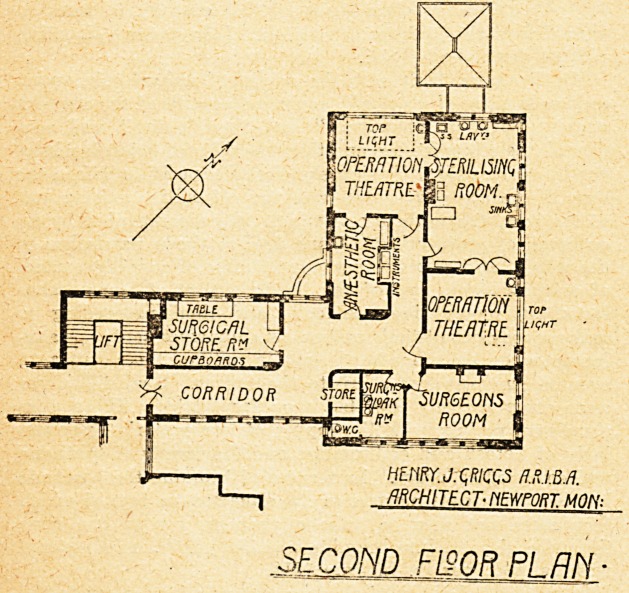


**Figure f3:**